# Book Reviews and Notices

**Published:** 1887-08

**Authors:** 


### BOOKS AND BOOK REVIEWS.

A TEXT-BOOK OF PATHOLOGICAL ANATOMY AND PATHOGENESIS. By ERNST ZIEGLER, Professor of Pathological Anatomy in the University of Tübingen. Translated by DONALD MACALLISTER, M.A., M. D. Three parts complete in one volume. Pp. xxvii. and 1091. NEW YORK: WILLIAM WOOD & Co. CHICAGO: W. T. KEENER. 1887.

This is apparently a reprint of the English edition in three volumes. It is a misfortune that the translator has omitted that part of the original German edition which treats of the pathological anatomy of the Bones, Muscles, Eye, Ear and Genital Organs, for, defend the omission as he may, the translated work remains incomplete and inferior to the original. Of the merit of the treatise it need only be said here that it is the most thoroughly systematic, complete and satisfactory text-book upon the subject in the English language. The idea of combining the three volumes in one is a good one both for convenience and cheapness, but it is a great pity that at the same time clear printing and clear cuts have been sacrificed. The printing is unworthy of the house which sends it out, and most of the cuts are very poorly engraved, two of them, numbers 74 and 80, being almost worthless. E. W.

A LABORATORY MANUAL OF CHEMISTRY, MEDICAL AND PHARMACEUTICAL. By OSCAR OLDBERG, Pharm., D., Professor of Pharmacy and Director of the Pharmaceutical Laboratories in the Illinois College of Pharmacy, Northwestern University, and John H. Long, Sc. D., Professor of Chemistry and Director of the Chemical Laboratories of the Chicago Medical College and the Illinois College of Pharmacy, Northwestern University. With original illustrations. pp. 445. Chicago: W. T. KEENER.

The object of the authors "in preparing this manual was to provide in convenient form a sufficent number of suitable lessons in laboratory work, and at the same time to embody in the book the facts of inorganic chemistry most important to pharmacy and medicine."

Part I. Describes the elementary substances, metallic and non-metallic, used in medicine and pharmacy, with experiments, tests and a list of the preparations of which they form a chief constituent.

Part II. "Synthetical Chemistry," describes inorganic compounds and compounds prepared with organic acids. The specific gravity, solubility and other ordinary chemical facts about these substances are given, and the method of preparation of the most of those that can be made in an ordinary chemical laboratory. In many cases the methods directed by several different pharmacopœias are given with criticisms as to their relative advantages.

Part III. "Analytical Chemistry," describes methods of qualitative analysis and volumetric analysis with sections on sugar analysis, determination of amounts of alcohol in fermented liquors, determination of diastatic power of malt extract, valuation of jalap, testing of pepsin, testing for some alkaloids as those of chinchona, opium, nux vomica, etc., and a chapter on urine-analysis.

The book is a laboratary manual and presupposes a considerable knowledge of the subject. Nothing is said of elementary or theoretical chemistry and very little about the numerous "dodges" which constitute so large a part of the manipulative skill necessary to results. But a teacher can explain these things quicker and better than any book. A solitary student, however, would often find himself at a loss in going through the exercises.

Occasionally the details in minor points are singularly redundant. Of iron we are told that "In the laboratory it is employed in stands, tongs, furnaces, oil and sand-baths, etc., on account of its strength and relative cheapness," etc., etc. Surely space might have been spared from this for a fuller description of methods.

The description of the various processes are, however, accurate and for the most part clear. An occasional obscurity detracts more from the symmetry than the usefulness of the book.

The chapter on urine-analysis is excellent. It does not contain many methods or those which, even if followed with great labor, would be of doubtful value; the best of the well tried ones are given; more would be an encumbrance to the student, or even to the practitioner, unless under exceptional circumstances.

The text is very free from printer's errors, a mark of great care in a book of this kind.

The cuts illustrate their subjects, though some might miss something in execution.

The plates of urine sediments cover most of the common forms, though room could have been spared for more, and all the possibilities of drawing, and especially of printing, were not exhausted in their production.

On the whole, the book is an excellent one and most of its faults will probably be removed in a second edition. LESTER CURTIS.

ON THE PATHOLOGY AND TREATMENT OF GONORRHŒA AND SPERMATORRHŒA. By J. L. MILTON, Senior Surgeon to St. John's Hospital for Diseases of the Skin, London. New York: WILLIAM WOOD & Co. 1887.

Mr. Milton has written a book, intended, so the preface informs us, as "one of reference for the busy practitioner."

Mr. Milton is an Englishman. He is pugilistic. His writing smacks of the same pugnacity that characterizes some of the prose works of his great namesake, the author of "Paradise Lost."

We imagine him detecting poor M. Chabalier in some error. He rubs his hands gleefully together, and calling on the "busy practitioners" to form a ring, says: "Now, gentlemen, though you are very busy and have come to consult me for another purpose, please pause a moment and see me polish off this insignificant Frenchman, who has attempted to tell what he knows about the history of gonorrhœa. See the foolishness of such a statement of this; the imbecility made manifest by this utterance; could any one with reason, with brains, have written this?" And so poor M. Chabalier leaves the ring with the black eye and bloody nose that Mr. Milton promised to give him.

Then the busy practitioners, hungry for information, crowd around Mr. Milton and ask him, as a man of learning and of high standing, to give them his opinion upon the evil effects of gonorrhœa, or upon his use of injections in the treatment of the disease. "That reminds me, gentlemen," he replies, "did you ever hear such nonsense as was uttered by Dr. Noeggerath, of New York? I have never seen his article upon the consequences of gonorrhœa, but I read a review of it once. If you will wait for a few moments until I show you how I can tear that article of Dr. Noeggerath's—well, ahem! I suppose I should say the review of the article, for, as I said, I have never seen the original—to pieces, I will gladly give you the result of my experience in treatment, etc." And there Dr. Noeggerath's article is torn to shreds. And so on through the book.

And Mr. Milton is not afraid. Such men as Hunter, Sydneham, Troussean, Cooper and Gross, have to take their share of the blows so freely given by this energetic and excitable Englishman. And many of the blows are fairly dealt. But the busy practitioner is supposed to consult Mr. Milton, not to witness his display of skill in demolishing theories and ideas, many of them dead and buried years ago, but to get Mr. Milton's own ideas; for every one knows that the opinion of a man of such wide experience and careful observation must be valuable. And if the practitioner has the time to read much that is not of practical value to him, he will find much that is of the greatest value. Moreover, Mr. Milton is always interesting. His literary style, especially in the line of sarcasm, is something not possessed by many medical writers.

Were Mr. Milton's chapter on spermatorrhœa let loose upon the general public, we fear it would cause consternation and fill the physicians' offices with anxious patients, if the laity suspected that a man of large experience and high standing in the profession held that every nocturnal emission was unnatural. A boy or man having more than one such emission a month should be treated. These views are at variance with those of one of our leading Chicago physicians, who being consulted by a young man for monthly nocturnal emissions startled him by exclaiming—"Great Heavens! only once a month! I'll try to give you something to make you have them once in two weeks anyhow" "Emissions are invariably more or less injurious." "The disease (spermatorrhœa) yearly reduces hundreds if not thousands to impotence and all its attendant ills, hypochondria, weariness of life, insanity and so on."

"I have passed the bougie hundreds of times for spermatorrhœa."

"The name (spermatorrhœa) has been objected to, and we have been told that the term ought to be applied to cases in which the semen trickles away insensibly from the urethra—a disease so rare that I have never seen a case, and strongly doubt the existence of it; to which the term spermatorrhœa is not a whit more suited than it is to emissions."

These are not quotations from quack's pamphlet, but from the volume on "Pathology and Treatment of Spermatorrhœa," by J. L. Milton, Senior Surgeon to St. John's Hospital for Disease of Skin, London.

WHAT TO DO IN CASES OF POISONING. By William Murrel, M. D., F. R. C. P. First American from the fifth English Edition. Edited by FRANK WOODBURY, M. D. PHILADELPHIA: THE MEDICAL REGISTER COMPANY. 1887.

This edition contains one hundred and fifty-eight pages. The author says that if there were fewer poisons the treatise would be smaller. The arrangement is into two general divisions, viz: "Introduction" which treats of the subject in a general way, and "The Poisons in Alphabetical Order," which treats of each in order under the topics, *How Taken*, *Symptoms*, *Fatal Dose*, *Treatment*. Both the arrangement and general treatment of the subjects leave nothing to be desired. In respect to the desirability of having this or some other equally good book upon the subject, the author's remarks about the stomach pump may be quoted. "Every doctor should have a stomach pump or an efficient substitute. It may not be wanted for years, but it may be wanted to-morrow, and a life, or many lives, may depend upon its being in working order." E. W.

THE NURSING AND CARE OF THE NERVOUS AND THE INSANE. By CHARLES K. MILLS, M. D. 12mo., pp. 147. PHILADELPHIA: J. B. LIPPINCOTT COMPANY. 1887. CHICAGO: W. T. KEENER.

This little work forms part of a series of "Practical Lessons in Nursing" now being issued by the Lippincott Company.

The first chapter deals with the management of the commoner nervous disorders and with the necessary qualifications, of a good nurse for nervous patients. The forms of insensibility are given and so much of their differential diagnosis as may be important for the nurse to know. On page 26 we find the old idea enunciated regarding the necessity of arousing the dormant nervous system, in cases of narcotic poisoning. Fortunately the old barbaric methods are not described. Chapter two relates to massage, muscle-beating, bathing, etc. This is much the most useful part of the work, and the most easily comprehended. Chapter three deals with the application of electricity, forms of batteries, etc. Here there is too much and yet not enough. We fancy the average nurse, who thoroughly masters this part of the work, will consider himself an accomplished electrician, and that too without just reason for the belief. With the varying forms of batteries and electrical appliances it is the duty of the physician, to direct the nurse just how a particular battery is to used and for how long in each case. As a matter of fact, the electrical treatment ought always to be given by the physician. The final chapter deals with the nursing and care of the insane, and is in the main a good exposition of the written and oral instruction given attendants in our better hospitals for the insane.

The work, as a whole, is not specific enough, nor is it simple in its explanations. Too much knowledge on the part of the reader is assumed. The physician and trained nurse will find many things of value in this work, but the nurse without previous elementary instruction will not find it to be of great value. H. N. M.

OUTLINES FOR THE MANAGEMENT OF DIET. By EDWARD TUNIS BRUEN. 12 mo., pp. 138. PHILADELPHIA: J. B. LIPPINCOTT COMPANY. 1887. CHICAGO: W. T. KEENER.

This third volume of the series of "Practical Lessons in Nursing," is a very creditable production. While the work claims to be but an outline of the subject, yet the rules for diet are concisely expressed and in sufficient fullness for all clinical purposes. Not only the nurse, but the physician as well, will find here valuable suggestions regarding diet in most of the cases for which he will be called upon to prescribe.

The work opens with a short description of the physiology of digestion. This is followed by an excellent chapter on the regulation of food in the different periods of life. The beverages as accessories of diet receive consideration, and the views adopted are similar in many respects to those held by Sir William Roberts. Special plans of diet are considered, and are followed by a chapter devoted to the amount of food required by the system, followed by one containing hints on the diet of the corpulent and the thin. The remainder of the work is devoted to the consideration of diet in special diseases.

The work is well arranged, well written, and forms a small but excellent addition to the literature of dietetics. H. N. M.

THE CURABILITY OF INSANITY AND THE INDIVIDUALIZED TREATMENT OF THE INSANE. By JOHN S. BUTLER, M. D. Pp. 59. NEW YORK and LONDON: G. P. PUTNAM'S SONS. CHICAGO: W. T. KEENER. 1887.

In this little volume Dr. Butler makes a strong and earnest plea in favor of the individual, *moral*, treatment of the insane. To secure this there must be more buildings, with fewer occupants to the building, segregation of patients. The man afflicted with acute melancholia must not be subjected to the depressing influences that overcome him, if associated daily with the one demented and beyond cure. The asylum must be, in fact as in name, a refuge, a retreat, a home for the insane, and not a prison where all who enter must lay aside all hope of leaving.

Dr. Butler believes that the present alarming increase in the number of insane is due to neglect of proper methods of prevention. The time to begin to prevent insanity is in childhood. Brains developed at the expense of the body, the pushing of the boy into business and the girl into society, are accountable for many of the wrecks of manhood and womanhood that our asylums can show. Not alone the alienist, but the gynæcologist, the oculist, the general practitioner as well, can cite instance after instance where careless or improper training of the child has led to irreparable trouble and disease.

Could this little essay be placed, where we fear it will not be, in the hands of parents, it would do much good. Yet, though the physician may not need argument to convince him of the strength of Dr. Butler's position, it will amply repay him to read the opinions of a man of wide experience and large heart. One leaves the book with the renewed determination to be more humane and gentle toward the unfortunate, and to do all in one's power to inculcate in the mind of the parent the importance of training children into men and women, well rounded out, physically and morally, as well as mentally and socially.

TRANSACTIONS OF THE PATHOLOGICAL SOCIETY OF PHILADELPHIA. Vol. XII. p. xxiv. and 289. Edited by W. E. HUGHES, M. D. PHILADELPHIA: Printed for the Society by W. J. DORNAN. 1886.

This volume contains the report of the proceedings of the society from September, 1883, to July, 1885. There are also given lists of the officers for each of the years mentioned above, of former presidents of the society and of the present members.

The arrangement is by systems, *e.g.*, The Vascular System, The Organs of Respiration, etc.

Many of the reports are of great interest and importance, but the limits of this notice will not allow special comment. Such publications as a whole are of very great value to the profession, and the society deserves warm commendation for its enterprise and liberality in printing and distributing the reports of its proceedings.

A TREATISE ON DIPHTHERIA, HISTORICALLY AND PRACTICALLY CONSIDERED: Including Croup, Tracheotomy and Intubation. By A. SANNE. Translated, annotated and the Surgical Anatomy added, by HENRY Z. GILL, A. M., M. D., LL. D. Pp. xxx and 656. ST. LOUIS: J. H. CHAMBERS & CO. 1887.

This translation is the work of an enthusiast, and he has evidently labored under the impression that the importance of the subject attaches equally to this particular book. As a whole it is comprehensive and complete. The style of the author, however, is prolix and discursive. The volume might be reduced one-half with advantage. The apology of the translator, in his preface, that he has been too busy to attend to his work is, at least, an unusual one. Despite these unfavorable features, he book is in many respects a good one, and may repay any who have patience enough to read it.

TREATMENT OF DISEASE IN CHILDREN, including the Outlines of Diagnonosis and the Chief Pathological Differences between Children and Adults. By ANGEL MONEY, M. D., M. R. C. P. Pp. xiii and 560, 16 mo. PHILADELPHIA: P. BLAKISTON, SON & CO. CHICAGO: W. T. KEENER. 1887.

It is a pleasure to commend such a book as this one is. The style of the author is very readable, and at the same time it is conservative, philosophic and able. He is the assistant physician to the Hospital for Sick Children, Great Ormond street, London, and what he has to say is evidently based upon a wide and ample experience. The chapters on "General Treatment of Disease," "The Principles and Practice of Feeding Infants and Children" are particularly good. That on "Diseases of the Nervous System" contains a discussion of "Raynaud's Disease," "Tetany," and much more not usually included in works upon disease in children. The table of contents and the index and the letterpress are good and the size of the book is very convenient. It can hardly fail to be one of the successful books. E. W.

THE PRACTITIONER'S HAND-BOOK OF TREATMENT: OR THE PRINCIPLES OF THERAPEUTICS. By J. MILNER FOTHERGILL, M. D. Pp. xx and 660, 16mo. Third American from the third English edition. PHILADELPHIA: LEA BROTHERS & COMPANY. 1887. CHICAGO: A. C. McCLURG & COMPANY.

The author repeats, in this edition, his statement that the book is not intended as a complete treatise on "Practice of Physic," but rather to aid the reader and to "guide him in his therapeutic evolution." The additions to this third edition are two chapters, one on "The Dietary in Acute Disease and Mal-Assimilation," and one on "The Management of Convalescence." For those unfamiliar with the author's writings it is only necessary to say that this book, like his others, while prolix, is very readable and contains much that is of great value. He tries to present his subject from a philosophical standpoint and succeeds admirably. He values accurate diagnosis as highly as any one, but does not deal with that subject in this work. E. W.

THE YEAR-BOOK OF TREATMENT FOR 1886: A Critical Review for Practitioners of Medicine and Surgery. Pp. viii., 304. PHILADELPHIA: LEA BROTHERS & CO. CHICAGO: W. T. KEENER.

This little book is issued annually for the purpose of furnishing a résumé of all the more important advances made in the treatment of disease during the year. Each department of practice is represented by a contribution from an author of note, who is specially interested in the subject upon which he writes. It should be purchased each year and read by all who are desirous of keeping abreast of the times, for in it will be found much of real value which by reason of the multitude of journals would otherwise have escaped attention. W. E. C.

LECTURES ON THE DISEASES OF THE NERVOUS SYSTEM. Delivered at the Hospital Salpêtrière. By J. M. CHARCOT, Professor of the Faculty of Medicine of Paris, Physician to the Hospital Salpêtrière, Member of the Institute and of the Academy of Medicine, Honorary President of the Anatomical Society, etc. Volume iii.

The third volume of Professor Charcot's most excellent work on the nervous system is a welcome addition to medical literature. The chief part of this volume is devoted to the study of hysteria in its many forms. Professor Charcot has written his ideas of hysteria in a clear and condensed form. He describes hysterical monoplegia, hystero-traumatic contractures, hysterical coxalgia, hysteria of boys and of men, hysterical mutism, rhythmical chorea of the hysterical, etc.

Considerable space is devoted to the amoytiophic disturbances following articular and chronic rheumatism. Finally, aphasia, in all of its forms, is treated in a clear and concise manner.

A MANUAL OF TREATMENT BY MASSAGE AND METHODICAL MUSCLE EXERCISE. By JOSEPH SCHREIBER, M. D. Translated by WALTER MENDELSON, M. D. Pp. viii and 285, large 16 mo. PHILADELPHIA: LEA BROTHERS & COMPANY. CHICAGO: A. C. MCCLURG & COMPANY. 1887.

The author gives Ling, the originator of the so-called "Swedish Movement Cure," great credit for his perseverance in establishing the system of treatment. He is in no sense a blind enthusiast and does not consider treatment by massage and exercise a panacea. He does think, however, that the method is capable of great usefulness when the cases for treatment are intelligently selected. He thinks that only very simple apparatus is necessary, but has profusely illustrated his text with apparatus which those may use who prefer it. The opening chapters treat the subject historically and generally, and others follow devoted to those diseases to which the treatment is adapted. The book, upon the whole, is methodical and thorough, and the style is attractive. E. W.

ANATOMICAL RESEARCHES IN THE HUMAN RECTUM AND A NEW METHOD OF RECTAL INSPECTION. By WALTER J. OTIS, M. D., of Boston. First part: The Sacculi of the Rectum, with one wood cut in the text and eight plates. [From PROFESSOR BRAUNE'S Department in the Anatomical Institute at Leipzig.] LEIPZIG: VEIT & COMPANY. 1887.

The first part is devoted to a careful description of the gross anatomy of the rectum as observed in studies made upon the cadaver. The writer dwells especially upon the conformation of the inner surface of the rectum as modified by the folds of mucous membrane encroaching upon the lumen of the tube, variously described by different authors. Also, upon the best methods of examining the rectum with the least distortion. For this he advises placing the patient in the lithotomy and knee-chest positiosn and the use of a retractor. The work is being published in German and English in parallel columns.

MATERNITY, INFANCY, CHILDHOOD. Hygiene of Pregnancy: Nursing and Weaning of Infants: The Care of Children in Health and Disease. By JOHN M. KEATING, M. D. Pp. 221, 12 mo. PHILADELPHIA: J. B. LIPPINCOTT & COMPANY, 1887, CHICAGO: W. T. KEENER.

This is one of the four books published by the same company in similar style, as a "Series" on "Practical Lessons in Nursing." The preface states that "this little work is intended for mothers and for those who have undertaken the care of infants and children in health and sickness." Probably no book on such a subject could be perfect. This one is at least very good and can be confidently recommended by physicians to those for whom it is intended. E. W.

